# Application of Biocides and Super-Absorbing Polymers to Enhance the Efficiency of Filtering Materials

**DOI:** 10.3390/molecules24183339

**Published:** 2019-09-13

**Authors:** Katarzyna Majchrzycka, Małgorzata Okrasa, Anita Jachowicz, Justyna Szulc, Bogumił Brycki, Beata Gutarowska

**Affiliations:** 1 Department of Personal Protective Equipment, Central Institute for Labour Protection—National Research Institute, 90-133 Łódź, Poland; kamaj@ciop.lodz.pl; 2 Institute of Fermentation Technology and Microbiology, Lodz University of Technology, 90-924 Łódź, Poland; anita.jachowicz@edu.p.lodz.pl (A.J.); justyna.szulc@p.lodz.pl (J.S.); beata.gutarowska@p.lodz.pl (B.G.); 3 Faculty of Chemistry, Adam Mickiewicz University in Poznań, 60-780 Poznań, Poland; brycki@amu.edu.pl

**Keywords:** bioactive filtering materials, biocides, super-absorbing polymers, survival of microorganisms, filtering efficiency, respiratory protection

## Abstract

Studies on the functionalization of materials used for the construction of filtering facepiece respirators (FFRs) relate to endowing fibers with biocidal properties. There is also a real need for reducing moisture content accumulating in such materials during FFR use, as it would lead to decreased microorganism survival. Thus, in our study, we propose the use of superabsorbent polymers (SAPs), together with a biocidal agent (biohalloysite), as additives in the manufacturing of polypropylene/polyester (PP/PET) multifunctional filtering material (MFM). The aim of this study was to evaluate the MFM for stability of the modifier’s attachment to the polymer matrix, the degree of survival of microorganisms on the nonwoven, and its microorganism filtration efficiency. Scanning electron microscopy (SEM) and Fourier transform infrared (FTIR) spectroscopy were used to test the stability of the modifier’s attachment. The filtration efficiency was determined under conditions of dynamic aerosol flow of *S. aureus* bacteria. The survival rates (N%) of the following microorganisms were assessed: *Escherichia coli* and *Staphylococcus aureus* bacteria, *Candida albicans* yeast, and *Aspergillus niger* mold using the AATCC 100-2004 method. FTIR spectrum analysis confirmed the pre-established composition of MFM. The loss of the active substance from MFM in simulated conditions of use did not exceed 0.02%, which validated the stability of the modifier’s attachment to the PP/PET fiber structure. SEM image analysis verified the uniformity of the MFM structure. Lower microorganism survival rates were detected for *S. aureus*, *C. albicans*, and *E. coli* on the MFM nonwoven compared to control samples that did not contain the modifiers. However, the MFM did not inhibit *A. niger* growth. The MFM also showed high filtration efficiency (99.86%) against *S. aureus* bacteria.

## 1. Introduction

Staff at many workplaces may be exposed to harmful aerosols [1,2,3,4,5]. A characteristic feature of biological hazards is their variability over time. In particular, pathogenic microorganisms undergo constant changes, evolution, and selection. For this reason, the correct selection of personal protection equipment (PPE) is an important issue because, very often, it is not possible to implement systemic solutions in the work environment. The use of filtering facepiece respirators (FFRs) as a protection against bioaerosols is common [6,7,8,9,10,11].

FFRs must meet a number of requirements directly linked to the safety of workers who use them. Most of all, the filtering nonwovens in FFRs must be able to efficiently retain microorganism particles. On the one hand, it depends on the properties of the nonwoven, while on the other, it depends on the properties of the polluted air flowing through the FFRs. In order to ensure high efficiency, FFRs should have low porosity, while at the same time have fibers with the least possible diameter (preferably below 1 μm) [12,13,14].

The second important parameter for assessing FFRs is whether microorganisms retained on the filtering material can survive. Published literature clearly shows that microorganisms grow on filtering polymer materials during use and storage [15,16,17,18,19,20,21,22]. This growth is mainly favored by the presence of moisture and organic compounds, e.g., dust particles [23,24], and can be detrimental to the user.

Several studies on endowing polymer fibers with biocidal properties have been conducted in order to counteract this negative phenomenon. Woo and colleagues published an example of a biocidal respiratory protective equipment [25]. Dialdehyde starch content of 4% in the filtering material caused an average 30% decrease in microorganism survival. Tiliket et al. published results describing the chemical modification of the cellulose fiber surface with polyethylenimine in order to obtain antiviral properties [26]. Likewise, Heimbuch and Wander used polistyren-4-methyltrimethylammonium triiodide (quaternary ammonium derivative of polystyrene in the form of triiodide) to impart biocidal properties to respiratory protective equipment [27]. They were able to reduce *E. coli* bacterial numbers by 99.99% on the surface of surgical masks. Majchrzycka et al. devised melt-blown nonwovens modified with a biocidal agent carried on perlite. The modifier particles were introduced into polypropylene fibers using melt-blown technology [28,29]. Gutarowska et al. took advantage of the commercial biocidal agent Sanitized^®^ and silver ions to modify filtering nonwovens. The researchers introduced these agents/ions onto finished nonwoven fabrics by dipping them into a bath or spraying [30,31]. Recent developments in biocidal materials include the temporal release of the biocidal agent and the use of gemini surfactants [3]. The latter are formed of two identical single-chain monomers connected by a linker between hydrophilic groups that ensures higher biocidal efficacy than classical biocides.

A new trend in the development of filtering nonwovens is to endow them with multiple functions by modifying with appropriate additives. There are physical and chemical methods of modification and their choice depends on the additive type. The technology for producing high efficiency filtering nonwovens (melt-blown) by introducing additives into semi-liquid polymer fibers stream deserves special attention. A method of introducing powdered additives has been developed, which overcomes the impact of the high temperature linked to the melting of the polymer matrix [28]. Using this method, we introduced two types of modifiers into the nonwoven structure: biocide and superabsorbent polymers (SAPs). This idea was based on results from earlier studies, which showed that moisture and dust favored microorganism growth on filtering materials used for the construction of FFRs [23,24].

There is growing interest in the use of SAPs for modifying the properties of filtering nonwoven materials used for specific technical applications. The most common examples include separating filters, retention fabrics, surgical dressings, food packaging, and electric cable cladding [32,33]. SAPs belong to a class of hydrophilic gels (hydrogels), i.e., macromolecular meshes of polymer chains that have the ability to absorb and retain water or aqueous solutions. SAPs absorb water through physical adsorption (water retention in micropores by capillary forces, hydrogen bonding of functional groups in polymer chains, and limited by crosslinking and expansion of polymer chains), which results in an increase in polymer volume.

The aim of our research was to assess the effectiveness of functional additives (biocidal halloysite (BH) and SAP) in the reduction of microorganisms’ survival on multifunctional filtering materials (MFM) used in FFRs. In addition, we also evaluated the effects that our modifications had on the stability of the fiber/additive structure, which is essential for safe use of these materials for respiratory protection.

## 2. Results and Discussion

### 2.1. Stability of the Modifier’s Attachment to the Fiber Structure

**Figure 1:**
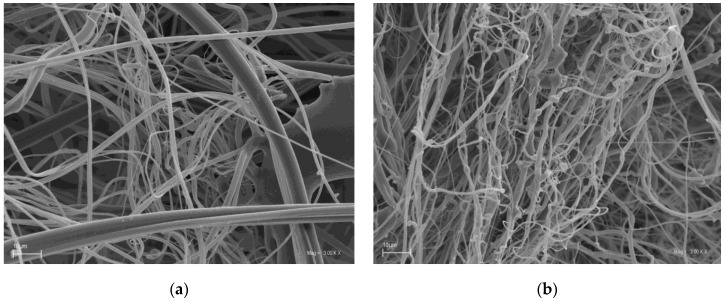
SEM images of (**a**) CS nonwoven (PP/PET) and (**b**) MFM nonwoven (PP/PET/SAP/BH).

Scanning electron microscopy (SEM) was employed to investigate the morphologies of MFM modified with SAP and BH (Figure 1b). For comparison, the SEM images of pristine PP/PET nonwovens (CS) were also investigated (Figure 1a).

SEM analysis confirmed that regardless of the qualitative composition, the filtering materials were characterized by a similar structure. No visible domains of condensed structure, which could reduce the stability of the material, were seen in the images. The analysis of the SEM images confirmed that the MFM had a homogeneous fibrous structure.

**Figure 2:**
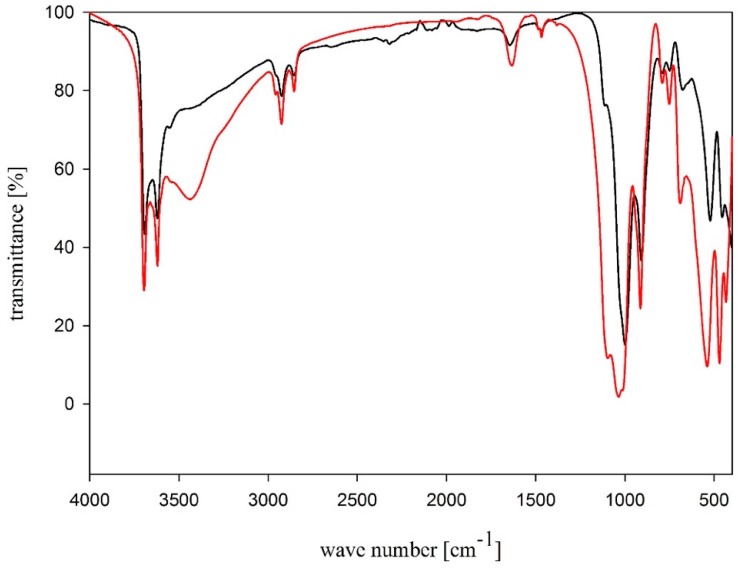
FTIR spectra of biohalloysite: ATR (black line), KBr (red line).

In order to confirm that SAP and biocidal halloysite (biohalloysite; BH) modifiers are incorporated into the MFM composition, FTIR analyses were performed. FTIR measured in KBr and attenuated total reflection (ATR) spectra of BH are shown in Figure 2.

Absorption bands at 3695 and 3620 cm^−1^ in the FTIR spectrum are related to the stretching vibrations of OH groups in the inner halloysite layer, whereas the OH deformation vibration band lies at 1650 cm^−1^. A broad, intensive band at 1100 cm^−1^ is linked to Si–O stretching vibrations, while a band at lower wavenumbers values, 1030 cm^−1^, corresponds to Si–O–Si stretching vibrations. The bands of symmetrical and asymmetrical stretching vibrations of CH_3_ and CH_2_ groups of the didecyldimethylammonium chloride alkyl chain lie in the range of 2962–2853 cm^−1^, while the deformation vibration band of CH_3_ and CH_2_ groups of the didecyldimethylammonium chloride alkyl chain lies at 1470 cm^−1^ [34,35,36,37,38].

**Figure 3:**
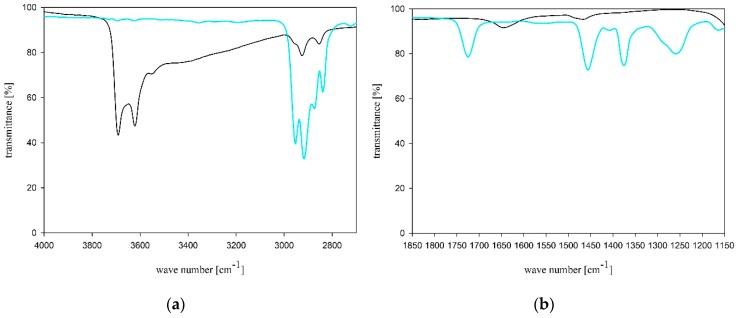
FTIR (ATR) spectra of BH (black line) and MFM nonwoven (blue line); (**a**) wave number range between 4000 and 2700 cm^−1^, and (**b**) between 1850 and 1150 cm^−1^.

FTIR (ATR) spectra for BH additive and MFM nonwoven are shown in Figure 3.

Due to a small addition of BH as a modifier of PP/PET nonwoven, the spectrum differences compared to CS were insignificant. However, there is a characteristic lack of absorption bands above 3620 cm^−1^ in the FTIR spectrum that originate from the OH group stretching vibrations in the inner halloysite layer. This is related to the loss of water molecules from the halloysite during MFM manufacturing using the melt-blown method. Noteworthy is the presence of the carbonyl group band characteristics for PET at 1725 cm^−1^. The tests confirmed the composition of MFM nonwoven.

The spectrophotometric quantitative analysis of quaternary ammonium salts or compounds (QACs) was used to test the stability of BH binding. This feature was determined based on the loss of didecyldimethylammonium chloride from the modified nonwoven. Based on the established standard curve, the concentration of didecyldimethylammonium chloride in the extract was determined. It was found that, under the conditions of extraction, in the first test (A) an unmeasurable amount of didecyldimethylammonium chloride underwent desorption, whereas in the second test (B), after 24 h, a loss of 0.019% was found. The loss of the active substance from the MFM filter material under simulated, extreme conditions did not exceed 0.02%, which would mean that the attachment is stable under normal conditions of use. Our analysis confirmed that the MFM nonwovens can be safely used for the construction of FFRs.

### 2.2. Survival of Microorganisms on Filtering Materials

Table 1 shows microorganism numbers on CS and MFM nonwovens at time points t = 0 and after 24 h of incubation. It also shows microorganism survival rate.

*E. coli* and *S. aureus* bacteria number on CS and MFM nonwovens at t = 0 ranged from 5.61 × 10^7^ to 2.75 × 10^8^ CFU/sample. The number of *C. albicans* and *A. niger* fungi was from 2.55 × 10^6^ to 5.99 × 10^6^ CFU/sample. There were no statistically significant differences between CS and MFM nonwovens at timepoint t = 0 (*p* < 0.05). Following 24 h of incubation, the number of bacteria increased and ranged from 1.09 × 10^7^ to 2.09 × 10^9^ CFU/sample. Similarly, the number of *C. albicans* yeast increased and ranged between 1.16 × 10^7^ and 2.48 × 10^7^ CFU/sample. In contrast, the number of *A. niger* mold reduced to between 1.35 × 10^5^ and 4.08 × 10^5^ CFU/sample. Microorganism numbers on the MFM nonwoven was lower compared to CS, which was statistically significantly for two of the four species studied, i.e., *S. aureus* and *C. albicans*. This shows an efficient inhibition of microorganism growth by nonwovens containing BH and SAP. This finding was further confirmed by survival rate values for *S. aureus* and *C. albicans* that were 7.5- and 2-times lower for MFM than for CS, respectively.

No statistically significant differences between the two types of nonwovens were observed for *E. coli* bacteria, while the survival rate was lower for the MFM nonwoven (N = 697) than for CS (N = 786). The number of *A. niger* mold and its survival rate were higher for MFM, which indicates a lack of activity of the nonwovens against this mold.

In the present study, inhibition of the growth of bacterial and yeast cells has been confirmed, which indicates biostatic activity of MFM nonwovens. Simultaneously, no significant antimicrobial activity (biocidal or biostatic) has been found for tested mold. Pankey and Sabath (2004) stated that clear distinction between biocidal and biostatic activity cannot be made. There are no antimicrobial agents that exclusively kill microorganisms, and others that only inhibit their growth. Showing differences between these categories of agents can be difficult and is often used arbitrarily, e.g., clinical antibacterial agents are described as potentially being both bactericidal and bacteriostatic [39].

In the current study, QACs, which are the modifier’s (BH) biologically active substance, were mainly responsible for the damage to the cell wall of the microorganisms and the resulting efflux of small-molecule intracellular components. This mechanism is not always effective at inhibiting microorganism growth, as it is largely dependent on their type. This is probably the reason for the differential efficiency of MFMs against the microorganisms used in our tests. According to the literature, the survival of microorganisms on filtering nonwovens differs by type of microorganism, physiology (including the ability to produce survival forms, i.e., endospores for Gram-positive rods and conidia for molds), and, most of all, cell wall structure [23,40,41]. The membranes of Gram-negative rods contain lipopolysaccharide (LPS) that protect the cell from biocides. In Gram-positive cocci, LPS is absent and the peptidoglycan layer forming the cell wall is a lot thinner [42]. Therefore, Gram-positive cocci are more sensitive to QACs than Gram-negative rods, as confirmed experimentally in the present work.

Gutarowska et al. studied antimicrobial action of filtering nonwovens containing QACs in the form of Sanitized^®^ T 99-19 biocide. Like in the current study, they found that bacteria were most sensitive, followed by yeast, while sensitivity was lowest for molds [30]. In addition, the authors considered various methods of manufacturing nonwovens (melt-blown, needle-punched), different methods of biocide application onto nonwovens (bath treatment, spraying), a range of biocide concentrations (from 2 to 7%), and conditioning of nonwovens as factors influencing antimicrobial efficacy. They showed that antimicrobial efficacy was greatest for the highest concentration of QACs used for needle-punched rather than melt-blown nonwovens, and for nonwovens subjected to bathing compared to spraying. It was demonstrated that thermal conditioning (70 °C or 30 °C for 24 h) had no effect on antimicrobial activity.

Micobiocide-N750-containing QACs were used as nonwoven modifiers in the studies by Gliścińska et al. [43]. They obtained high antimicrobial activity against *S. aureus* and slightly lower for *E. coli* at a biocide concentration of 1.5%, which is consistent with the results presented in this study. However, the authors did not appraise the effectiveness of the nonwovens against fungi.

Majchrzycka et al. analyzed polypropylene melt-blown filtering nonwovens used in FFRs modified with a biocide based on alkylammonium QACs delivered on two mineral carriers (bentonite and perlite) [29]. The research showed that a concentration of 10% of the biocidal agent on a mineral carrier was sufficient to inhibit the growth of *E. coli* and *S. aureus*. This study also did not evaluate the effectiveness of nonwoven fabrics against fungi.

To date, no bioactive filtering nonwoven containing SAP for the construction of FFRs has been developed. The results obtained by Majchrzycka et al. show that within 7 min from the start of FFR use, there is a rapid increase of humidity to 92% at a temperature of 29–30 °C, which are favorable conditions for bacterial growth. Under model conditions, it was demonstrated that the survival of *S. aureus* was the highest, lower for *E. coli* and *C. albicans*, and insignificant for *B. subtilis* bacteria and *A. niger*. Therefore, the addition of SAP to the filtering nonwovens to limit moisture, which promotes bacterial growth, is justified. The results of the antimicrobial activity of MFM nonwovens against the tested microorganisms are satisfactory, as they limit the growth of those microorganisms (*S. aureus*, *E. coli*, *C. albicans*) that have the greatest ability to grow on FFRs under conditions simulating real-world use. It is possible to utilize a higher biocide concentration to increase the effectiveness of MFMs against molds. However, based on earlier studies, controlling this group of microorganisms on FFRs is not justified. Non-sporing bacteria and yeasts have the highest survival rates on FFRs; therefore, limiting the growth of these groups of microorganisms is crucial.

### 2.3. Filtration Efficiency against Microorganisms

While assessing filtration efficiency, it is important to select the correct dimensions of bioaerosol particles, as the results strongly depend on the dimensions of particles retained by the fibers. Smaller particles are retained to a lesser degree compared to larger ones, e.g., droplets formed during coughing, sneezing, and speaking (ca. 20 µm) [40,41]. For this reason, the assessment of filtration efficiency was carried out for *S. aureus* bacteria, whose average diameter is 1 µm. This is comparable to the droplet size of *Mycobacterium tuberculosis* and *Bacillus anthracis* spore, as confirmed by environmental studies [42,44]. The results of tests on filtration efficiency of SC and MFM are presented in Table 2.

Filtration efficiency determined for CS and MFM against *S. aureus* bacteria was high: 99.86% and 99.96% for MFM and CS, respectively, for particles of 1 µm. According to the theory of filtration, such particles are retained on the fibers by diffusion. Random movement of air molecules collide with these small particles and cause them to wander across a stream of air until they come in contact with a fiber. Much smaller particles of the order of 0.1 μm, characteristic of viruses, are also retained by the same mechanism. These include Adenovirus causing respiratory infections, Filoviruses responsible for the onset of Ebola hemorrhagic fever, Orthomyxoviridae causing types A, B, and C influenza, and Coronaviridae causing SARS. However, it has been shown that there are significant differences in evaluating the filtration efficiency for 0.1 and 1 μm particles [45,46], which shows that the high MFM filtration efficiency achieved can only be applied to bacteria. From a practical point of view, MFMs may constitute the base material for FFRs intended to protect the respiratory system against bacteria, i.e., *Mycobacterium tuberculosis* and *Bacillus anthracis*.

MFMs may also be used as a base material for so-called “surgical respirators”, types of surgical masks which help prevent the spread of infection from the wearer’s exhaled breath to potentially susceptible persons [47]. Surgical masks are used, as a norm, to prevent nosocomial infections, but only act as a barrier for large droplets of liquids. Surgical respirators, on the other hand, are generally used when it is necessary to protect the respiratory system of medical staff. Their filtration efficiency tends to be much higher than that of surgical masks. They should be able to block large droplets expelled by the wearer and efficiently retain small particles of the order of 1 µm inhaled from the environment. An additional requirement for surgical masks is leaktightness of fit on the wearer’s face [48]. This parameter is closely related to the shape of the facepiece of the half mask and the harness system. MFMs meet the above requirements for high filtration efficiency (99.86% for particle size of 1 µm). For technical reasons, it is possible to develop MFMs that have differing surface mass, but maintain high particle filtration ability (thin fibers and additional electrification process). This makes it possible to produce diverse shapes of the half mask cup.

Standard FFR tests, in accordance with local regulations in different parts of the world, are always conducted using model aerosols, which are so-called physical penetration agents. Likewise, it is also important to test the filtering materials used in the construction of FFRs, in accordance with such regulations, as well as determine filtration efficiency against microorganisms—the so-called viable penetration. Although it is not a standard test, it should be performed as it has been proven that there may be statistical differences between physical and viable penetration [8]. In this case, the results of assessing filtration efficiency depend on test conditions, i.e., flow, humidity, and, most of all, the characteristics of the microorganisms (size, spheres, rods, and rod/sphere shape). When comparing particles of the same aerodynamic diameter, spherical particles always penetrate more through the filtering nonwoven compared to particles of a different shape.

Filtration efficiency also depends on the characteristics of the filtering material (mostly fiber thickness), the air flow rate, electrostatic charge of the particles being filtered, and the charge of the material accumulated on the fibers of the filter. The bioaerosol flow rate in the study was set to 30 L/min, which corresponds to the air flow rate of FFRs, while performing medium–hard work.

Both types of filtering materials (CS and MFM) had the same electrostatic potential because, during their production using the melt-blown technique, the final process involved subjecting them to corona discharge with a negative voltage of 30 kV. This strengthens the filtering mechanism by imparting the materials with electrostatic attraction in addition to diffusion. This may have contributed to the high filtration efficiency of CS and MFM against *S. aureus* bacteria. It can be concluded that the modifiers with biocidal and superabsorbent properties added to PP/PET did not affect the degree of fiber electrification, which is evidenced by the lack of statistical differences between the filtration efficiency of CS and MFM materials.

Eninger et al. presented the results of studies on filtering nonwovens sampled from FFRs, including iodinated polymer with antimicrobial properties [49]. The efficiency testing against biological particles was conducted with the MS2 bacteriophage virus. It was determined that the FFR’s filtering material without the biocidal agent had a 95–98% filtration efficiency, while an increase in filtration efficiency was seen for the iodinated polymer. According to the authors, this was due to the densification of the filtering material structure with an iodine resin powder. A similar phenomenon was not seen for MFMs. This may be due to a low concentration of the modifiers and their fragmentation; 4 g of BH and 3 g of SAP were added per sheet of MFM nonwoven. Considering the MFM surface mass was 130 g/m^2^, and the fiber thickness ca. 1 µm (shown in Figure 1), with uniform distribution of the modifier, this conclusion is highly probable.

## 3. Materials and Methods

### 3.1. Nonwoven with Functional Modifiers

Two types of polypropylene filtering nonwovens manufactured using melt-blown technique were used within the study: (i) pristine polypropylene-poly(tereftalan ethylene) nonwoven (Control Sample; CS) and (ii) bioactive super-absorbing polypropylene-poly(tereftalan ethylene) nonwoven (Modified Filtering Nonwoven; MFM). Experimental work was conducted using a single-screw laboratory extruder. The temperatures of the heating zones of the extruder ranged from 250 to 345 °C, and the polymer melt was blown with hot air stream at a temperature of 310 °C. For MFM nonwovens, 4 g per sheet of biohalloysite (BH) and 3 g of super-absorbing polymer (SAP) per sheet were added, using a specially constructed pneumatic nozzle located in the fiber-forming head channel, directly into the fiber-forming zone, bypassing the high temperature zone [28,29]. After leaving the nozzle, BH and SAP particles hit the stream of semi-liquid polymer fibers so that they could be permanently fused to the polymeric material. Resulting nonwovens (CS and MFM) had an average surface mass of 130 g/m^2^ and average thickness of 2.1 mm.

Commercially available superabsorbent polymer (EK-X, Nippon Shokubai, Japan) and BH (95% halloysite/5% didecyldimethylammonium chloride *wt*/*wt*) were used as modifiers of the polymer filtering materials. BH was obtained by the deposition of didecyldimethylammonium chloride on halloysite nanotubes. The use of didecyldimethylammonium chloride (CAS 7173-51-5, WE 230-525-2) as an active substance with antimicrobial activity is authorized by European Union Parliament and Council regulation (EU) No 528/2012 of 22 May 2012, which concerns market availability and use of biocidal products.

### 3.2. Assessing the Stability of the Modifier’s Attachment to the Fiber Structure

Microscopic evaluation was conducted for the two types of material, i.e., CS and MFM. SEM images (3000× magnification) were obtained from four samples of both the obverse and reverse sides of each material.

Using spectrophotometric quantitative analysis of QACs (UV), we assessed the loss of didecyldimethylammonium chloride from BH-modified PP/PET fibers over the time period simulating FFR use.

Two samples of the MFM filtering material, with a weight of ca. 20 g, were weighed with an accuracy of 0.0001 g and subjected to extraction in 750 cm^3^ of demineralized water (conductivity <10 μS at 36 ± 2 °C) for 15 min (test A) and 24 h (test B). At the end of the extraction, the aqueous solution was quantitatively transferred into a 1000 cm^3^ graduated flask and the UV absorbance was measured with methyl orange according to methodology reported in the literature [50,51,52].

### 3.3. Survival of Microorganisms on Filtering Materials

*Escherichia coli* (ATCC 10536), *Staphylococcus aureus* (ATCC 6538), *Candida albicans* (ATCC 10231), and *Aspergillus niger* (ATCC 16404) microorganisms stored at the Pure Culture Collection ŁOCK 105 of the Institute of Fermentation and Microbiology were used for the evaluation of microorganism survival on CS and MFM nonwovens. Statistical quantitative method from AATCC 100-2004 “Antimicrobial Finishes of Textile Materials” was used for determining microbial survival.

In order to obtain inocula of bacteria and yeast, 40 mL of sterile Tryptic Soy Broth (TSB, Merck, Darmstadt, Germany) medium for bacteria and Malt Extract Broth (MEB, Merck, Darmstadt, Germany) medium for yeast were inoculated with collection strains. The inocula were incubated at either 37 °C (bacteria) or 30 °C (yeast) for 24–48 h. Mold inoculum was obtained by washing spores with sterile MEB medium off Malt Extract Agar (MEA, Merck, Darmstadt, Germany) medium containing a 5-day *A. niger* culture. Optical density of inocula is shown in Table 3.

Square nonwoven swatches of 4 cm^2^ (2 × 2 cm) were cut from sheets of CS and MFM; 0.1 mL of bacterial or fungal inoculum was placed onto them. Following inoculation, samples were placed in sterile Petri dishes and incubated in Binder-720 climatic chamber (temperature T = 30 ± 2 °C; relative humidity RH = 80%).

CS and MFM samples were analyzed immediately after inoculation (t = 0) and after 24 h of incubation. Samples were transferred into plastic containers containing 50 mL of sterile 0.85% (normal) saline solution and shaken for 5 min. Next, serial dilutions were performed in normal saline solution and 1 mL or 0.1 mL of appropriate dilutions were seeded onto sterile Petri dishes, flooded with Tryptic Soy Agar semi-solid medium (TSA, Merck) for bacteria and MEA for fungi. The plates were incubated at 37 °C (bacteria) for 24–48 h or at 30 °C for 72 h (fungi), and the colonies grown were counted (the result was given in colony-forming unit per 4 cm^2^ area sample; CFU/sample). The studies of microorganism survival on multifunctional composite were carried out in two independent replicates.

### 3.4. Microorganism Filtering Efficiency

*Staphylococcus aureus* (ATCC 6538) bacterium stored at the Pure Culture Collection ŁOCK 105 of the Institute of Fermentation and Microbiology was used for the evaluation of filtration efficiency of CS and MFM nonwovens.

In order to obtain *S. aureus* bacterial inoculum, 600 mL of sterile TSB were inoculated. The resulting inoculum suspension was incubated at 37 ± 2 °C for 24–48 h. The number of bacteria in the inoculum suspension was determined by culture method. Serial dilutions were performed in saline solution and flooded with semi-solid TSA microbiological medium. Following an incubation at 37 ± 2 °C for 24–48 h, colonies were counted, considering the dilution and volume of the seeded inoculum. The density of inoculum suspension was 1.61 × 10^8^ ± 4.27 × 10^7^ CFU/mL.

**Figure 4:**
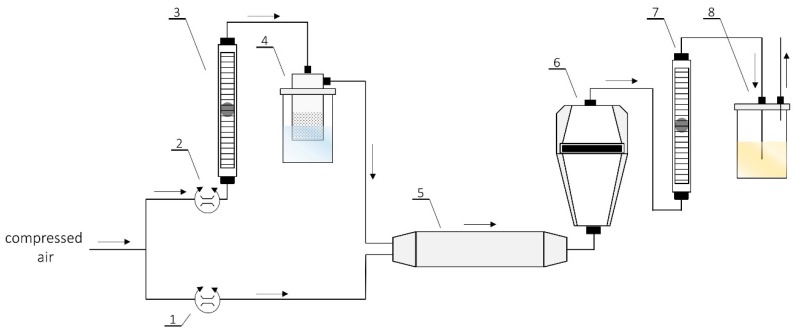
The scheme of the set-up used for the studies of efficiency and biocidal effectiveness of nonwovens: (**1**,**2**) Valves for precise air flow regulation compressor; (**3**,**7**) air flow meter; (**4**) collison atomizer; (**5**) in-line desiccator; (**6**) test chamber; and (**8**) outlet of bioaerosol.

*S. aureus* bacterial suspension was used as the bioaerosol, which was passed through the filtering nonwovens (CS, MFM), and the microbiological filter was set up based on the schematic shown in Figure 4. Bacterial cultures were centrifuged at 5000 rpm for 10 min in order to separate the cells from the medium. The medium was removed, whilst bacteria were suspended in 600 mL of sterile normal saline solution. Bacterial suspension was mixed on a magnetic stirrer; all steps were performed under aseptic conditions. The suspension was transferred into a sterile container-atomizer and connected to the equipment.

The research was performed in a chamber with laminar air flow equipped with HEPA filters and a UV lamp. The tests consisted of dynamic generation of bioaerosol by an atomizer, which was mixed with a stream of dry air. This was subsequently directed into a system of filters installed in a tightly sealed setup. The flow rate of the bioaerosol was 30 L/min and was controlled by a system of rotameters. Next, in each test, a two-filter system was fixed: (I) a filter made of multifunctional composite, and (II) a microbiological filter. The microbiological filter was used for quantitative analysis of the number of bacteria retained on the filtering material. An 80 mm gelatin microbiological filter (Sartorius, Göttingen, Germany) with a 3 µm pore size and 99.9999% retention rate was used for the study. A control experiment to determine the number of bacteria in 1 m^3^ of bioaerosol generated under experimental conditions (flow rate of 30 L/min over 20 min, which corresponds to passing 600 L of bioaerosol through the sample) was also performed. The number of bacterial cells in the bioaerosol amounted to 4.98 × 10^8^ CFU/m^3^.

The equipment was switched on and the bacterial aerosol was passed through the sample for 20 min each time. CS and MFM samples were analyzed immediately after bioaerosol inoculation (time t = 0) and following 24 h of incubation at 37 °C. The samples were placed into plastic containers filled with 50 mL sterile normal saline and shaken for 5 min. Subsequently, serial dilutions of the samples were performed in normal saline solution (from 10^−3^ to 10^−9^) and 1 mL or 0.1 mL of appropriate dilutions were seeded onto sterile Petri dishes and flooded with semi-solid TSA medium. Plates were incubated at 37 ± 2 °C for 24–48 h, and the resulting colonies were counted (the results were given as CFU/sample). The experiments were conducted in two independent replicates for each variant examined.

The second microbiological filters used in the bioaerosol passing system were transferred into sterile saline in 50 mL plastic containers, dissolved, serially diluted, and seeded on TSA medium according to the procedure described above.

### 3.5. Mathematical Analysis

Following the Grubbs test and after rejecting uncertain results, three results were selected for statistical analyses. The arithmetic means and standard deviations of the number of microorganisms grown on plates were calculated using Microsoft^®^ Excel. Differences between the numbers of microorganisms on CS and MFM samples were analyzed using One-Way Analysis of Variance (ANOVA). Differences were considered significant at *p* < 0.05. All data were analyzed using the Origin 6.1 software (OriginLab Corporation, Northampton, MA, USA).

Microorganism filtration efficiency was calculated as a ratio between the number of microorganisms retained on the microbiological filter (N_f_) and the sum of all microorganisms present in the system during test. Microorganism numbers on CS and MFM (N_w_) were calculated as the difference between the number of all microorganisms present in the system during tests and the number of microorganisms retained on the microbiological filter. Filtration efficiency was expressed as a percentage.

Microorganism survival rate (N) on CS and MFM following 24 h incubation was calculated according to the formula:

N = (N_t_/N_0_) × 100% (1)

where N_0_ is the number of microorganisms present on the filtering nonwoven at t = 0 [CFU/sample], while N_t_ is number of microorganisms present on the filtering nonwoven after incubation t = 24 h [CFU/sample].

## 4. Conclusions

Lower microorganism numbers and lower microorganism survival rates were found for MFM compared to CS in three out of four species studied, i.e., *S. aureus*, *C. albicans*, and *E. coli.* Furthermore, the MFM showed high filtration efficiency (99.86%) against *S. aureus* bacteria. FTIR analysis of MFMs confirmed the pre-established composition of the modified filtering nonwovens. The loss of the active substance from MFMs, in simulated conditions of use, did not exceed 0.02%, which confirmed the stability of the attachment of modifiers to the structure of PP/PET fibers. SEM analyses confirmed the uniform fibrous structure of the MFM.

Our research contributes to expanding the range of potential applications of functional modifiers with biocidal and superabsorbent activity in products intended to protect people against airborne hazards at work. Of particular interest is the use of BH and SAP in filtering nonwovens, more so because there is an increased interest in using such additives to personalize textile products and improve their hygienic properties.

## Figures and Tables

**Table 1:** Bacteria number, survival rate, and antimicrobial activity of nonwovens.

Microorganism	Nonwoven Type	Microorganism Numbers on Nonwovens [CFU/Sample *]		Survival Rate, %
		at 0 h	after 24 h	
*E. coli*	CS	M: 2.66 × 10^8^	M: 2.09 × 10^9^	786
		SD: 1.10 × 10^8^	SD: 2.96 × 10^8^	
	MFM	M: 2.75 × 10^8^	M: 1.92 × 10^9^	697
		SD: 8.50 × 10^7^	SD: 2.07 × 10^8^	
*S. aureus*	CS	M: 5.61 × 10^7^	M: 6.04 × 10^8^	1076
		SD: 1.07 × 10^7^	SD: 1.03 × 10^8^	
	MFM	M: 7.61 × 10^7^	M: 1.09 × 10^8^ ^a^	143
		SD: 3.91 × 10^7^	SD: 7.91 × 10^7^	
*C. albicans*	CS	M: 5.94 × 10^6^	M: 2.48 × 10^7^	417
		SD: 8.32 × 10^5^	SD: 6.28 × 10^6^	
	MFM	M: 5.99 × 10^6^	M: 1.16 × 10^7^ ^a^	193
		SD: 8.82 × 10^5^	SD: 2.66 × 10^6^	
*A. niger*	CS	M: 2.55 × 10^6^	M: 1.35 × 10^5^	5
		SD: 3.19 × 10^5^	SD: 8.26 × 10^4^	
	MFM	M: 2.80 × 10^6^	M: 4.08 × 10^5^ ^a^	15
		SD: 4.97 × 10^5^	SD: 9.88 × 10^4^	

M, mean value; SD, standard deviation; * sample of surface area of 4 cm^2^; CS, PP/PET electret melt-blown nonwoven (control sample); MFM, PP/PET/SAP/BH multifunctional electret melt-blown nonwoven; ^a^ significantly different number of microorganisms on MFM from the CS (One-Way ANOVA, *p* < 0.05).

**Table 2:** CS and MFM filtration efficiency against *S. aureus* bacteria.

Nonwoven	Bacteria Number on Microbiological Filter [CFU/Sample *]	% of Bacteria Retained by the Microbiological Filter	% of Bacteria Retained on Filtering Materials
CS	M: 1.26 × 10^5^ SD: 4.92 × 10^4^	0.042	99.96
MFM	M: 4.33 × 10^5^ SD: 3.12 × 10^5^	0.140	99.86

M, mean value; SD, standard deviation; * microbiological filter of 50.24 cm^2^ surface area dissolved in 50 mL of sterile normal saline solution; number of *S. aureus* cells in the system 2.99 × 10^8^ CFU/20 min at 30 L/min flow; CS, PP/PET electret melt-blown nonwoven (control sample); MFM, PP/PET/SAP/BH multifunctional electret melt-blown nonwoven.

**Table 3:** Density of inoculating solutions of microorganisms.

Microorganisms	Inoculum Density [CFU/mL]
*E. coli*	M: 2.65 × 10^9^
	SD: 4.12 × 10^8^
*S. aureus*	M: 2.64 × 10^8^
	SD: 1.20 × 10^8^
*C. albicans*	M: 5.33 × 10^7^
	SD: 4.72 × 10^6^
*A. niger*	M: 2.48 × 10^7^
	SD: 5.50 × 10^6^

M, mean value; SD, standard deviation
